# Shared Mechanisms Drive Ocular Following and Motion Perception

**DOI:** 10.1523/ENEURO.0204-24.2024

**Published:** 2024-06-14

**Authors:** Philipp Kreyenmeier, Romesh Kumbhani, J. Anthony Movshon, Miriam Spering

**Affiliations:** ^1^Department of Ophthalmology & Visual Sciences, University of British Columbia, Vancouver, British Columbia V5Z 3N9, Canada; ^2^Graduate Program in Neuroscience, University of British Columbia, Vancouver, British Columbia V6T 1Z3, Canada; ^3^Center for Neural Science, New York University, New York, New York 10003; ^4^Department of Psychology, New York University, New York, New York 10003; ^5^Institute for Computing, Information, and Cognitive Systems, University of British Columbia, Vancouver, British Columbia V6T 1Z3, Canada; ^6^Djavad Mowafaghian Center for Brain Health, University of British Columbia, Vancouver, British Columbia V6T 1Z3, Canada

**Keywords:** eye movements, ocular following, pattern motion, perception–action

## Abstract

How features of complex visual patterns are combined to drive perception and eye movements is not well understood. Here we simultaneously assessed human observers’ perceptual direction estimates and ocular following responses (OFR) evoked by moving plaids made from two summed gratings with varying contrast ratios. When the gratings were of equal contrast, observers’ eye movements and perceptual reports followed the motion of the plaid pattern. However, when the contrasts were unequal, eye movements and reports during early phases of the OFR were biased toward the direction of the high-contrast grating component; during later phases, both responses followed the plaid pattern direction. The shift from component- to pattern-driven behavior resembles the shift in tuning seen under similar conditions in neuronal responses recorded from monkey MT. Moreover, for some conditions, pattern tracking and perceptual reports were correlated on a trial-by-trial basis. The OFR may therefore provide a precise behavioral readout of the dynamics of neural motion integration for complex visual patterns.

## Significance Statement

Navigating our natural environment requires that we sense, perceive, and track the motion of objects. Here we investigate how pattern motion signals are computed and integrated to drive human eye movements and perception. We show that ultrashort latency ocular following movements and perception integrate complex motion signals similarly, shifting from component-driven to pattern-driven responses during the first fraction of a second after the onset of visual motion. These results resemble the shift in tuning in neuronal responses recorded from monkey MT and indicate that human ocular following provides a precise behavioral readout of these neuronal dynamics.

## Introduction

The primate visual system analyzes motion in two stages. First, orientation- and direction-selective neurons in the primary visual cortex (V1) signal the velocities of single components within a pattern. Second, component velocity signals are integrated to compute the true motion of objects and patterns. The perceptual and motor impact of these two stages of motion processing can be conveniently studied using two-dimensional plaid patterns, created by summing one-dimensional gratings of different orientation (Adelson and Movshon, 1982). When the grating components of a plaid have equal contrast, human observers perceive the coherent motion veridically. But when one component is higher in contrast, the perceived motion is biased toward it (Stone et al., 1990; Yo and Wilson, 1992; Bowns, 2013). This form of motion integration may reflect the activity of neurons in the middle temporal visual area (MT; Movshon and Newsome 1996; Rust et al., 2006), a main motion processing hub for perception and oculomotor control (Newsome et al., 1985; Newsome and Paré, 1988; Dürsteler and Wurtz, 1988; Kawano, 1999; Lisberger and Movshon, 1999; Takemura et al., 2007; Ilg and Thier, 2008). Recording experiments in MT revealed “component cells” that are tuned to the direction of single plaid components and “pattern cells” that integrate component signals and are tuned to the true direction of a plaid (Movshon et al., 1985; Rodman and Albright, 1989; Smith et al., 2005; Rust et al., 2006). When plaid components have different contrasts, the true motion of the pattern is unchanged, but the direction perceived by human observers and tuning of MT pattern cells both shift toward the direction of the higher-contrast component (Stone et al., 1990; Kumbhani et al., 2008).

We wondered how the signals that give rise to the perceptual experience of motion are related to those that drive motion-dependent eye movements. The eye movement we chose is the ocular following response (OFR)—a short-latency eye movement evoked by the onset of large-field visual motion (Miles et al., 1986; Gellman et al., 1990; Masson and Perrinet, 2012). With a latency of 70–85 ms in humans (Gellman et al., 1990), 55–60 ms in monkeys (Miles et al. 1986), and 38–53 ms in marmosets (Pattadkal et al., 2023; Yip et al., 2023), the OFR provides a direct, behavioral readout of early visual motion processing (Sheliga et al., 2005; Miles and Sheliga, 2010; Masson and Perrinet, 2012; Sheliga and FitzGibbon, 2023) and of motion integration over time (Masson and Castet, 2002). We simultaneously recorded human perceptual direction estimates and OFR in response to plaids composed of gratings with varying luminance contrasts and presented for different lengths of time. Similar contrast-dependent pattern sensitivity in eye movements and perceptual reports would be congruent with psychophysical studies concluding that eye movements and perception rely on shared processing of direction and speed signals (Gegenfurtner et al., 2003; Stone and Krauzlis, 2003; Maechler et al., 2021; for reviews, see Schütz et al., 2011; Spering and Montagnini, 2011). Alternatively, visual signals may be processed differently for eye movements and perception (Spering and Gegenfurtner, 2007; Tavassoli and Ringach, 2010; Spering et al., 2011; Simoncini et al., 2012; Glasser and Tadin, 2014; Lisi and Cavanagh, 2015; for a review, see Spering and Carrasco, 2015). Whereas most of these studies compared perception and eye movements that are under cognitive control (saccades and smooth pursuit), we compared perception to reflexive, short-latency OFR to assess whether perception and eye movements share early visual motion processing.

Our results show that perceptual reports of briefly presented motion and the earliest OFR are biased by the high-contrast component of the plaid. With increasing presentation duration and during later OFR and continuous tracking, perceptual reports and the OFR shift toward the direction of the pattern. During these late phases, perceptual and oculomotor performances are correlated on a trial-by-trial basis. This suggests a tripartite link between visual motion perception, oculomotor control, and the cortical machinery of motion processing.

## Materials and Methods

### Observers

We collected data from eight observers (mean age, 28 years; five females, one author). Observers had normal or corrected-to-normal visual acuity and no history of ophthalmic or neurological disorders. The University of British Columbia Behavioral Research Ethics Board approved all experimental procedures, which were in accordance with the Declaration of Helsinki. Observers gave written informed consent and received a remuneration of 10 CAD/h.

### Apparatus and stimuli

We conducted the experiment in a dimly lit laboratory. Observers viewed stimuli binocularly on an 18″ (1,280 × 1,024 pixels; 85 Hz) gamma-corrected cathode–ray tube monitor (ViewSonic G90fB) placed at a viewing distance of 50 cm at which the screen subtended 38.7° (horizontal) × 30.9° (vertical). A PC running MATLAB R2019b (MathWorks) and Psychophysics (version 3.0.12; Brainard, 1997; Pelli, 1997) and EyeLink toolboxes (Cornelissen et al., 2002) controlled stimulus presentation and data acquisition. We recorded the position of the right eye with an EyeLink 1000 tower-mount eyetracker (SR Research) at 1,000 Hz.

**Figure 1.**
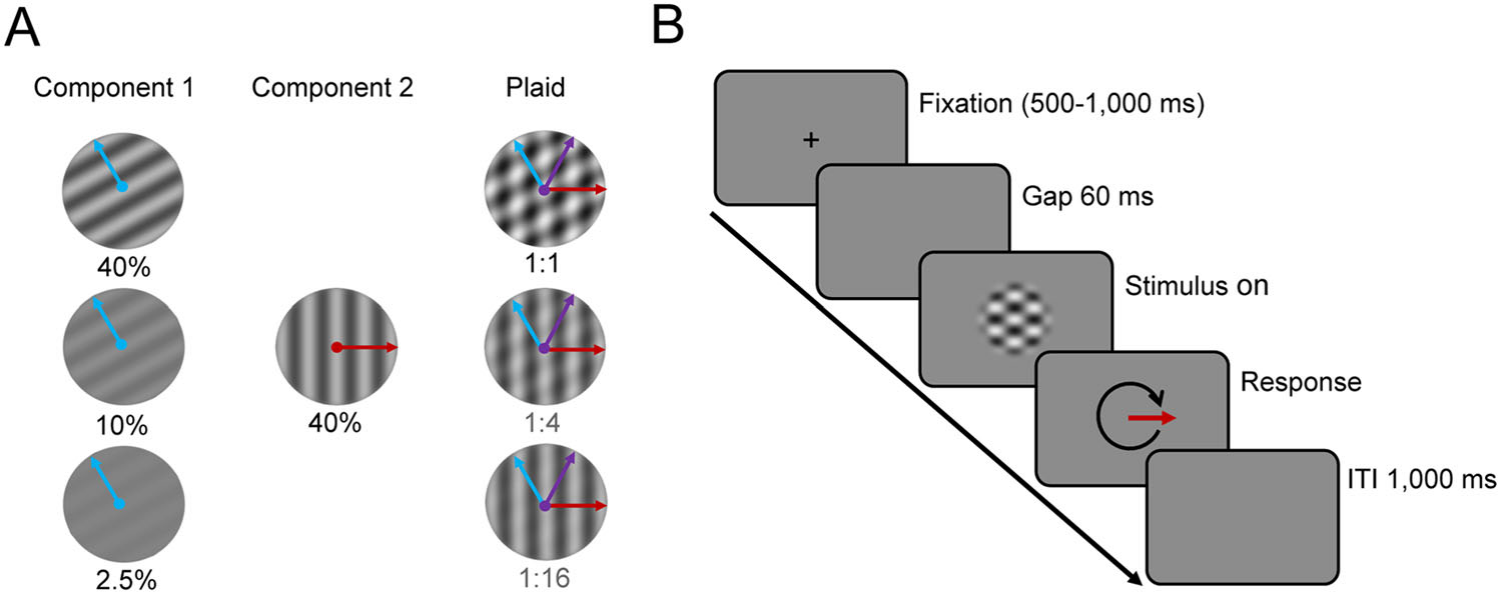
***A***, Example plaids with a 1:1, 1:4, and 1:16 contrast ratio made by adding two gratings with different orientations and contrasts. Blue and red arrows indicate component motion directions; the purple arrow shows pattern direction. ***B***, Trial timeline.

We compared perceptual estimates of motion direction with eye movements elicited in response to briefly presented gratings or plaids shown within a circular aperture subtending 20° and vignetted by applying a mask with smoothed edges. The gratings and plaids had the same mean luminance as the background (61.7 cd/m^2^). Plaids were made by adding two drifting sinewave gratings whose motion directions, always orthogonal to their orientations, differed by 120°. We varied the Michelson contrast of one grating between 2.5 and 40% in octave steps while holding the contrast of the other (high-contrast) grating constant at 40% to create five different plaids with contrast ratios 1:16, 1:8, 1:4, 1:2, or 1:1. We also presented a single, high-contrast grating (40% contrast). To eliminate the influence of any potential direction-dependent anisotropies in OFR magnitudes (Gellman et al., 1990), the motion direction of the high-contrast component (Fig. 1*A*, red arrow) was randomly selected between 1° and 360° for each trial; the other component always moved in a direction 120° counterclockwise (Fig. 1*A*, blue arrow). The grating spatial frequency was 0.25 cycles/degree, and the drift rate is 6 Hz, yielding a speed of 24°/s for each component grating and 48°/s for the plaid.

### Experimental design

At the beginning of each trial, observers fixated a centrally located cross for a randomly selected time between 500 and 1,000 ms. Following a 60 ms gap, we presented a drifting plaid or grating for a randomly chosen duration of 70, 130, 250, or 500 ms. At the end of each trial, observers indicated the perceived stimulus direction by rotating an arrow on the screen using a trackball mouse. The next trial began after an intertrial interval of 1,000 ms (Fig. 1*B*). Randomly interleaved catch trials (1/7 of trials) contained no stimulus, and observers pressed the mouse button to continue to the next trial. We instructed observers to pay close attention to the stimulus, but they received no explicit eye movement instruction.

The computer selected plaid contrast ratio, presentation duration, and motion direction randomly for each trial. Each experimental session consisted of 420 trials, separated into five blocks of 84 trials each. Observers performed three 1 h sessions over 3 separate days. Across sessions, each observer completed a total of 1,260 trials, resulting in 45 repetitions per condition [45 × 7 (five plaids with different contrast ratios + high-contrast grating + catch trial) × 4 (presentation durations) = 1,260].

### Eye movement recording and data preprocessing

We analyzed eye movements within a time window of −50 to 500 ms from the motion onset. We obtained eye velocity by differentiating eye position signals over time and smoothing using a second-order Butterworth filter (40 Hz cutoff frequency).

We removed saccades from eye movement traces for the OFR analysis by replacing values with NaNs. Saccades were defined as samples in which eye velocity exceeded a fixed velocity threshold of 30°/s for a minimum of 5 ms; the nearest reversal in eye acceleration before eye velocity exceeded the threshold marked the saccade onset, and the nearest reversal after eye velocity returned below threshold marked the saccade offset.

For analysis and data presentation, we calculated the relative eye direction with respect to the motion direction of the high-contrast component. To this end, we rotated the data so that the high-contrast component motion was always at 0° (represented as horizontal). With the low-contrast component oriented 120° counterclockwise relative to the high-contrast component, it follows that the rotated pattern motion direction (defined as the intersection of constraints; Adelson and Movshon, 1982) was always at 60° counterclockwise relative to the high-contrast component (Fig. 1*A*, purple arrows). We refer to the 0° (high-contrast component) direction as “component,” the 90° direction as “orthogonal,” and the plaid direction (60°) as “pattern.”

We detected the mean OFR onset by calculating least-squares fits of a two-segment piecewise linear function to the 2D eye velocity traces; the time of the break point defined the onset. Due to the low signal-to-noise ratio during the early phase of the OFR, we detected OFR onsets based on mean eye velocity traces across all trials per condition and observer rather than for individual trials (Yip et al., 2023). We manually inspected all trials and excluded those with blinks, those in which the tracker lost the signal, or trials with undetected saccades within the first 300 ms of the stimulus onset (3.1% of all trials). We also excluded trials in which observers reported a direction >60° from either the component or pattern direction (3.2% of all trials). One observer reported the opposite direction to the component or pattern motion in 19.5% of their trials. These trials were inconsistently spread across contrast ratios, presentation durations, and sessions; we excluded them from analysis. After applying the above exclusion criteria and eliminating catch trials, 8,123 trials of 10,080 trials remained for analyses.

### Perceptual data and statistical analyses

**Figure 2.**
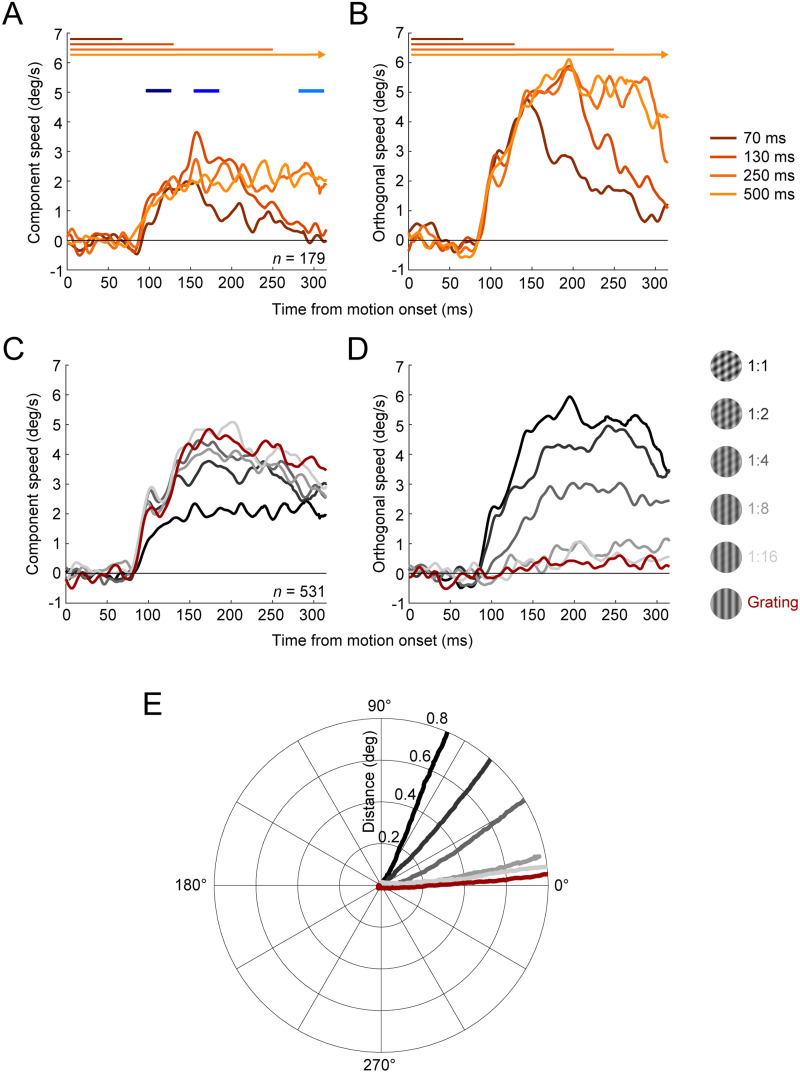
Single observer data, rotated as described in Materials and Methods so that eye movements in the direction of the high contrast component are labeled as component and eye movements 90° away as orthogonal. ***A***, Component and (***B***) orthogonal speed over time for the 1:1 contrast ratio. Traces show averages across trials for each presentation duration. Blue lines indicate analysis time windows for early OFR (dark blue, 95–135 ms), late OFR (medium blue, 155–195 ms), and tracking (light blue, 275–315 ms). ***C***, Component and (***D***) orthogonal speed for each contrast condition (gray shades; red line indicates grating), for the two longest duration conditions. ***E***, Average eye movement trajectories for each contrast ratio.

We rotated observers’ perceptual judgments in the same way as their eye movements (i.e., treating 0° as the component direction and 60° as the pattern motion direction) and collated responses for trials of each duration (70, 130, 250, and 500 ms). For the first three of these, we then compared perceptual responses with the direction of the OFR measured in 40 ms time windows chosen to begin 25 ms after the stimulus offset: 95–135 ms after the stimulus onset (“early OFR”), 155–195 ms (“late OFR”), and 295–315 ms (“tracking”; Fig. 2*A*); we also collected behavioral reports after the longest trials, for which there was no corresponding OFR measurement due to an increased number of saccades and a decrease in eye velocity during the later tracking response. To quantify pattern sensitivity for OFR and perception, we calculated contrast ratio functions. We computed least-squares fits to the perceptual and eye movement data of the following equation:

(1) $$\Theta (r)=\Theta_{\max}\frac{r^{n}}{r^{n}+r_{50}^{n}},$$

where Θ denotes the angle of the reported or eye movement direction, Θ_max_ the direction of the true motion of the stimulus (60°), *r* the contrast ratio, and *r*_50_ the contrast ratio at half asymptotic direction (30°). We used the *r*_50_ parameter to compare sensitivity to pattern motion direction between OFR and perception. Lower *r*_50_ values indicate higher sensitivity to pattern motion direction, and higher values indicate a bias toward the high-contrast component.

We used single-trial estimates of reported and tracked direction to ask whether OFR and perception were correlated on a trial-by-trial basis. For this analysis, we applied an exclusion criterion so that only trials in which the eyes moved at a velocity of at least 1°/s and within 60° of either the component or pattern direction. We ran three linear mixed models (one per OFR analysis time window) with eye movement (early OFR, late OFR, or tracking), contrast ratio, their interaction, and observer as a grouping variable, allowing random intercepts and slopes for the correlation between eye movements and perception. All statistical analyses were conducted with an alpha level of 0.05, unless otherwise stated.

## Results

We recorded observers’ eye movements and motion direction estimates in response to drifting gratings or plaids of different contrast ratios. Figure 2 shows eye speed responses in the component (Fig. 2*A*) and orthogonal motion directions (Fig. 2*B*) for the observer with the strongest OFR (highest peak speed). This observer tracked stimuli at a latency of 83 ms after the stimulus motion onset. For the shortest presentation duration (Fig. 2*A*,*B*, dark orange lines), OFR speed peaked at ∼150 ms and then started to decay. Speed peaked at ∼200 ms and then decayed for the 130 ms condition or was maintained for at least 320 ms for the two longest presentation durations (light orange lines). Based on these and similar traces for other observers, we chose to use data for all presentation durations for the early OFR measurements, data for the three longest durations for the late OFR measurements, and data for the two longest durations for the tracking measurements. Figure 2, *C* and *D*, shows component and orthogonal speed for the different contrast ratios for the same individual observer. Plots show averages across trials for the two longest presentation durations. When presented with a single, high-contrast grating (Fig. 2*C*,*D*, red curve), this observers’ eyes moved solely in the component direction (zero orthogonal speed, Fig. 2*D*). With increasing contrast of the second component, component speed decreased, and orthogonal speed increased, indicating a shift toward the pattern direction. Transforming 2D eye positions into polar coordinates (Fig. 2*E*) confirmed that the eyes moved in the 0° direction in response to single gratings and for plaids with large contrast ratios of component contrasts. The OFR was driven toward the pattern motion direction when component contrasts were within a factor of 2.

### Similar pattern motion sensitivity for OFR and perception

**Figure 3.**
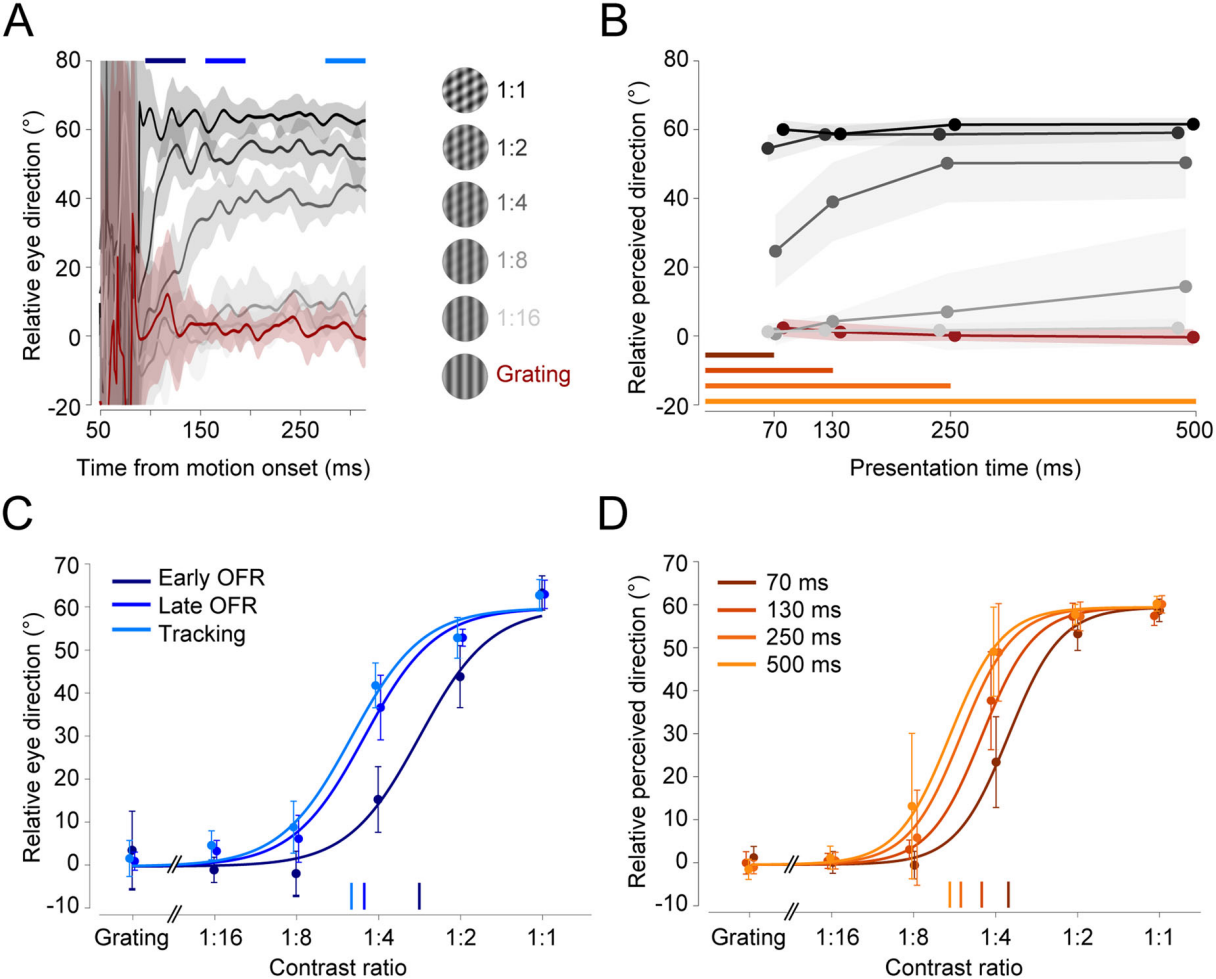
Comparison of contrast-dependent biases in OFR and perceptual estimates across observers (*n *= 8). ***A***, Eye movement direction over time relative to the stimulus onset for each contrast ratio. Traces show averages across observers and shaded areas represent ±1 SD. ***B***, Reported motion direction for each contrast ratio and presentation duration. Shaded areas represent ±1 SD. ***C***, Contrast ratio functions for early (dark blue), late (medium blue), and tracking (light blue) phases of the OFR. Dots and error bars show mean ± 1 SD across observers. Vertical lines on the baseline indicate *r*_50_. ***D***, Contrast ratio functions for perceived direction for each presentation duration. Same conventions as in panel ***C***.

Eye movement responses were consistent across observers and closely resembled the results for the single observer shown in Figure 2. Across all observers, OFR was initiated with a mean latency of 80 ms (±4 ms). Figure 3*A* shows eye direction relative to the component direction (0°) over time across all observers. Before the OFR onset (<80 ms), the eyes moved in random directions. Around the time of the OFR onset, variability in eye movement direction decreased, and the range of tracked directions collapsed to values between 0° (component direction) and 60° (pattern motion direction), depending on contrast ratio. When presented with a single, high-contrast grating or a 1:16 plaid, the eyes moved in the direction of the high-contrast component. When both components had the same contrast (1:1 plaid), the eyes followed the pattern motion. For intermediate contrast ratios (1:8, 1:4, and 1:2), the eyes followed a weighted average of the two components. For these intermediate contrast ratios, the OFR direction shifted over time: during the early OFR (Fig. 3*A*, dark blue), eye movements were biased toward the high-contrast component—most pronounced for plaids with contrast ratios of 1:4 and 1:2 (Fig. 3*A*, darker shades of gray). During the late OFR and during tracking, eye movements were less biased toward the high-contrast component and more closely followed the pattern motion. This indicates a shift in the OFR from an early, component-driven response to a later, pattern-driven response that reflects a contrast-dependent weighted average of the directions of the two plaid components.

Presenting stimuli for different durations allowed us to analyze perceptual reports as a function of time. Each data point in Figure 3*B* corresponds to one presentation duration. These results show that observers perceived component motion for the grating and the 1:16 plaid and pattern motion for the 1:1 and 1:2 plaids. For intermediate contrast ratios, observers’ perception was biased by the high-contrast component when the stimulus was presented for 70 ms but shifted toward pattern motion for longer presentation durations, in alignment with OFR results.

To directly compare pattern motion sensitivity between perceptual estimates and OFR, we plotted the tracked and reported direction as a function of contrast ratio and then fit contrast ratio functions. We extracted the contrast ratio at 50% of pattern direction (*r*_50_) as an indicator of pattern motion sensitivity. Figure 3*C* shows best-fit contrast ratio functions for different phases of the OFR, using the means across all observers. A leftward shift of the function along the horizontal axis implies increasing pattern sensitivity from early to late OFR to tracking. We observed a similar increase in pattern sensitivity with increasing presentation duration for perceptual reports (Fig. 3*D*).

**Figure 4.**
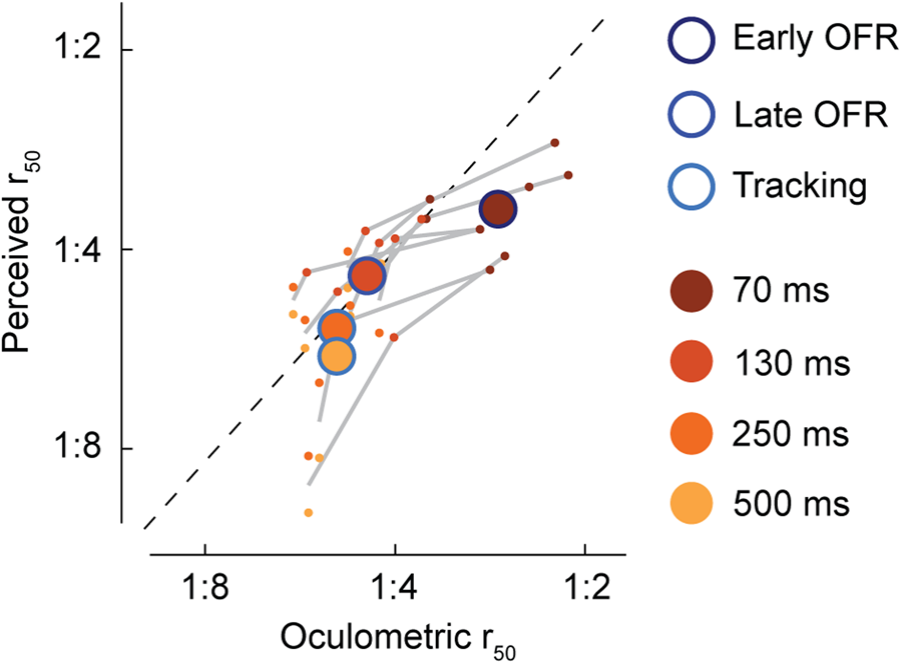
Comparison of biases in perception and tracking. Orange colors denote presentation durations; blue outlines are OFR phases. Large circles are averages across observers per comparison between perception and tracking (e.g., dark orange circle with dark blue outline shows comparison between the shortest presentation duration and the early OFR phase). Gray lines connect individual observer data and show consistent time-shifted sensitivity across observers. Note, because we compare perceptual responses for the two longer presentation durations to the same tracking response, gray lines connect the average of the two longer presentation durations with the individual data points for the shorter presentation durations.

Figure 4 shows results of a condition-by-condition comparison of pattern motion sensitivity in perceptual reports versus the OFR. A linear mixed model with observer as a grouping variable and random effects intercept and slope yielded a significant regression model (*β *= 0.673; *t*_(9.61)_ = 6.42; *p *< 0.001), confirming a similar shift from early, component-driven to later, pattern-driven responses in eye movements and perception.

Comparing *r*_50_ values between the early phase of the OFR and perceptual reports in the shortest presentation duration condition revealed that observers’ earliest perceptual reports were more sensitive to pattern motion than the early OFR, indicated by lower *r*_50_ values for perceived direction (Fig. 4, dark red dots). For all later analysis windows and longer presentation durations, pattern sensitivity was comparable between OFR and perceptual estimates (Fig. 4, yellow and orange dots). Across all conditions, the exponents of the best-fit contrast ratio functions were higher for perception (M* *= 5.92; SD = 1.54) compared with eye movements (M* *= 3.55; SD* *= 0.42; *t*_(7) _= 4.02; *p *= 0.005), indicating higher sensitivity in perception than in eye movements.

### Common noise sources for OFR and perception

**Figure 5.**
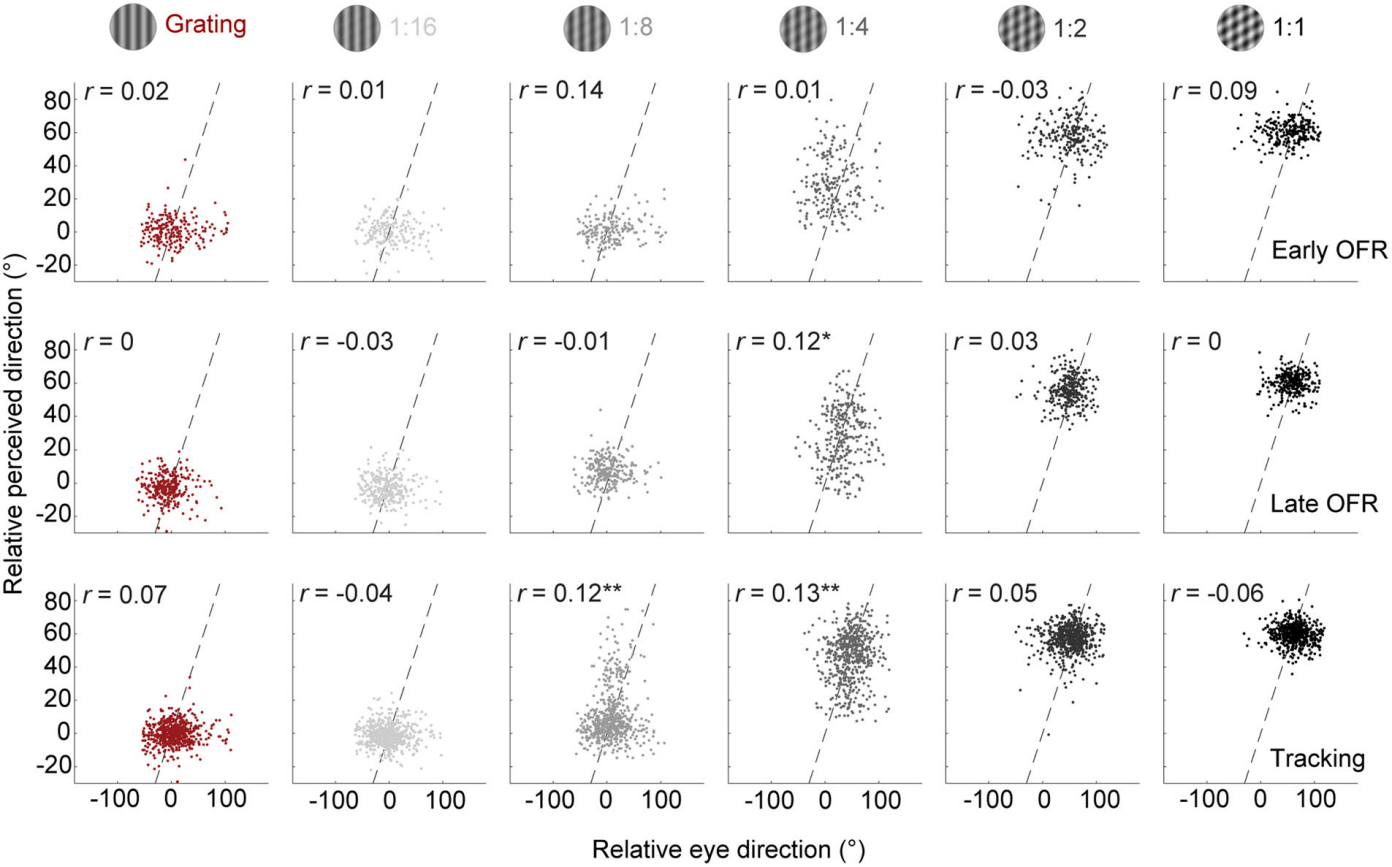
Trial-by-trial correlations between perceptual reports and OFR for all observers. Top row, early OFR (95–135 ms); middle row, late OFR (155–195 ms); bottom row, tracking (275–315 ms). Data for the tracking phase were pooled from the 250 and 500 ms presentation times. Dashed lines are identity lines.

We next analyzed the relationship between eye movements and perception on a trial-by-trial basis to determine whether variability in the OFR and in perceptual reports relies on common or private noise sources. Pooling data from all observers and trials for contrast ratios and OFR analysis intervals revealed larger variability in the OFR than in perceptual reports by a factor of 3. Within-subject variability was M* *= 37.4 (SD* *= 6.9; reported as across-subjects mean) for early OFR (Fig. 5, top row), M* *= 27.3 (SD* *= 5.1) for late OFR (middle row), and M* *= 28.8 (SD* *= 5.4) for tracking (bottom row). As Figure 5 reveals, variability was much smaller and largely constant across presentation durations for perceptual reports, ranging between M = 10.5 and 11.5.

In line with our observation that eye movements and perception showed similar pattern motion sensitivity during the later analysis window and longer presentation times, we observed trial-by-trial correlations between perceptual reports and OFR during the late tracking phase (Fig. 5, bottom row). Correlations were only observed for intermediate contrast ratios. We did not observe correlations at high- and low-contrast ratios, likely due to ceiling or flooring effects, respectively, with little to no variability in reported directions. These results were confirmed by linear mixed models for each analysis window, yielding a significant interaction between contrast and tracking only for the third analysis interval (*F*_(5,3475.73)_ = 7.15; *p *< 0.001). Whereas these results indicate that OFR and perceptual estimates might share the same sensory noise during later tracking and longer presentation times, we did not find significant correlations during the early OFR. A correlation during this early phase might be obscured by the large variability and low signal-to-noise ratio during early OFR.

## Discussion

We studied how the relative contrast of plaid components affects OFR and pattern motion perception and report three main results. First, plaids composed of gratings with equal luminance contrasts elicited an OFR in the direction of the pattern, aligned with observers’ perceived motion direction. For plaids with varying contrast ratios, we found a gradual shift in OFR direction tuning: early OFR was biased toward the high-contrast component, whereas late OFR corrected the initial bias and moved the eyes toward a weighted average of the two plaid components, with weights determined by contrast sensitivity. This indicates a shift in motion signal integration over time, from an early, component-driven response to a later, pattern-driven response (Pack et al., 2004; Smith et al., 2005). Second, perceived motion direction was biased by the high-contrast component when the stimulus was presented briefly but shifted toward pattern motion for longer presentation durations to become aligned to the direction of late OFR and tracking. Third, for intermediate contrast ratios, eye tracking and perceptual estimates were correlated on a trial-by-trial basis.

### Shared pattern motion signals in eye movements and motion perception

Perception and tracking eye movements rely on motion processing in middle temporal (MT) and medial superior temporal areas (MST) (Newsome et al., 1985; Dürsteler and Wurtz, 1988; Newsome and Paré, 1988; Kawano, 1999; Lisberger and Movshon, 1999; Takemura et al., 2002, 2007; Ilg and Thier, 2008). A subset of MT neurons signals the direction of pattern motion (Movshon et al., 1985; Rodman and Albright, 1989; Smith et al., 2005; Rust et al., 2006). These pattern-sensitive cells form the neural basis of coherent pattern perception (Adelson and Movshon, 1982; Movshon et al., 1985). When the components in a plaid have different luminance contrasts, human observers’ perception is biased toward the higher-contrast component (Stone et al., 1990; Yo and Wilson, 1992; Bowns, 2013). A contrast-dependent perceptual bias is congruent with findings showing that sinusoidal grating contrast modulates perceived speed (Thompson, 1982) and the velocity gain of smooth pursuit eye movements (Spering et al., 2005). Contrast modulation of encoded component speed would result in contrast-dependent weighting when combining grating components to compute pattern motion direction (Stone et al., 1990). Indeed, the tuning curves of MT pattern cells in macaque monkeys are biased toward the higher-contrast component when responding to plaids composed of different contrast components (Kumbhani et al., 2008). Using the same plaid stimuli as Kumbhani and colleagues, we have now demonstrated a strong contrast-dependent bias in the earliest phase of the OFR, which gradually shifts toward the pattern motion direction for the late OFR and which matches perceptual reports.

Previous studies revealed that the direction of early OFR depends on the relative contrast of the single components within a complex moving image (Sheliga et al., 2006, 2020). In a series of experiments, these authors investigated OFR in response to a square-wave grating that lacks the fundamental Fourier component. When shifting the stimulus one-fourth wavelength forward, it contains components that appear to move either forward or backward. First, Sheliga et al. (2005) found that the early OFR was elicited in the backward direction of the moving stimulus (in accordance with the backward direction of the major Fourier component of the stimulus, the third harmonic). This finding reveals that the OFR is sensitive to the Fourier composition of the moving stimulus, suggesting that the OFR provides a behavioral readout of first-order motion processing. Second, when reducing the contrast of the third harmonic, the direction of the OFR reversed its direction and was primarily driven by the next dominant Fourier component (which moved in the forward direction; Sheliga et al., 2006). Interestingly, a relatively small difference in contrast between components was sufficient to reverse OFR direction, indicating that the early phase of the OFR is driven by a nonlinear, contrast-weighted summation of component responses (Sheliga et al., 2020). Our findings of an early bias toward the high-contrast component fit well with these previous observations. Adding to this literature, we also found that the early component-driven response shifted toward a pattern-driven response over time. Using plaids composed of one drifting and one stationary grating (Type 2 or unikinetic plaids), Masson and Castet (2002) also observed an early component-driven response in the direction of the drifting grating and a subsequent pattern-driven response in the OFR. These behavioral observations are consistent with neurophysiological studies. Pack and Born (2001) measured the direction tuning of MT neurons to bar textures moving 45° obliquely to their orientation. Neural activity in area MT was initially dominated by component motion (or “contour”) signals orthogonal to the bars. Over time, direction preference shifted and exhibited the true pattern (or “terminator”) motion direction (Lorenceau et al., 1993). Parallel findings and a rapid transition from component to pattern cell activity were observed with unikinetic plaids (Wallisch and Movshon, 2019). This change in direction tuning from component to pattern motion could be due to a combination of slower processing of low-contrast pattern (terminator) components (Majaj et al., 2002) and to a longer latency of pattern signals in MT (Pack et al., 2004; Smith et al., 2005). In our situation, a late-arriving pattern motion signal would help the oculomotor system to align the late phase of the OFR with pattern motion.

### Common and private sensory noise sources for OFR and perception

**Figure 6.**
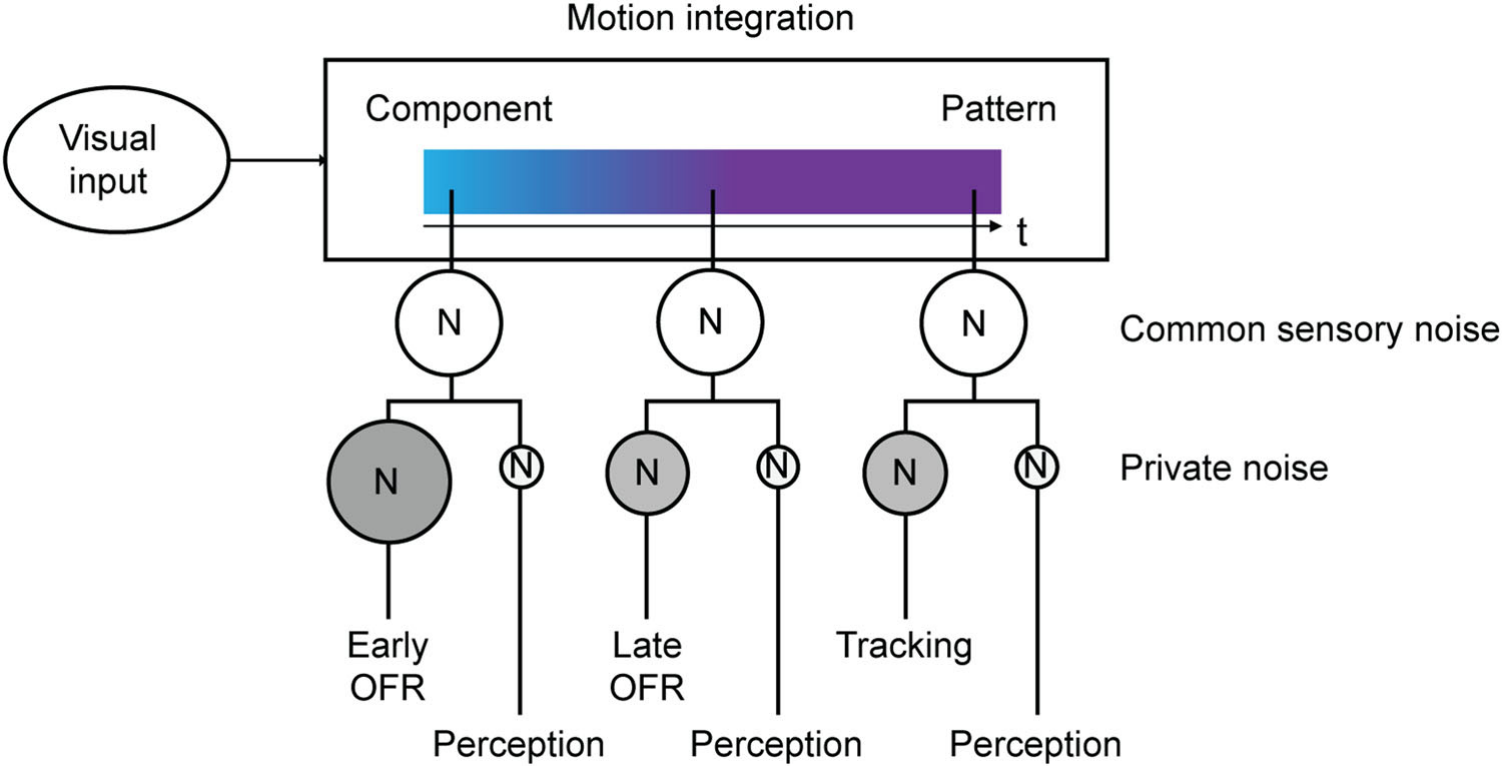
Schematic diagram summarizing key findings and proposed mechanisms. Plaids and gratings are processed via a motion integration stage providing a shared pattern motion signal for OFR and perception. Two noise sources are proposed: a common sensory noise source shared by perception and eye movements and a private source for each effector system. N, noise; size of the circles illustrates magnitude of intrasubject trial-by-trial variability.

We observed trial-by-trial correlations between OFR and perception during the later tracking phase and for longer presentation times. Congruent with these findings, human smooth pursuit eye movements and perceptual direction judgments covary on a trial-by-trial basis (Stone and Krauzlis, 2003). Monkeys’ smooth pursuit variability during the initiation phase (first 140 ms of the response) is largely due to sensory noise that might be shared with perceptual responses (Osborne et al., 2005, 2007). Based on these findings, variability in eye movements and perception may result from a shared sensory noise source, with private noise sources specific to the perceptual and eye movement system added further downstream (Stone and Krauzlis, 2003; Lisberger, 2010). Figure 6 shows how this model may account for our results. Our data suggest that overall, OFR and perception rely on a similar readout of pattern motion signals. The computed signal is time-dependent and gradually shifts from a component-driven to a pattern-driven response (Pack and Born, 2001; Smith et al., 2005). Sensory noise may be shared by both effector systems, and additional, effector-specific noise sources may act downstream. In line with our finding that trial-by-trial variability was larger for eye movements compared with perception, we propose that a private noise source underlies the variability in eye movements, which may include motor noise and larger measurement noise. We did not observe a correlation between OFR and perception for the early analysis window and the shortest presentation time. On the one hand, this may be the result of different motion processing for perception and early OFR (Simoncini et al., 2012), in line with our finding that perceptual reports were more sensitive to pattern motion than early OFR. On the other hand, the higher trial-by-trial variability during early OFR, compared with later OFR and tracking, might have masked a correlation between eye movements and perception. During this early phase, eye speed is slow and prone to low signal-to-noise ratio.

## Conclusion

Our natural environment often contains complex motion patterns that must be captured and encoded by our visual and oculomotor systems. To understand how we operate in this challenging visuomotor world, it is critical to understand how pattern motion signals are computed to drive eye movements and perception. Here we show that ultrashort latency ocular following movements and perception integrate complex motion signals similarly, shifting from component-driven to pattern-driven responses during the first fraction of a second after the onset of visual motion. This also demonstrates that the OFR provides a sensitive and noninvasive tool to probe early visual motion processing (Sheliga et al., 2005; Miles and Sheliga, 2010; Masson and Perrinet, 2012; Sheliga and FitzGibbon, 2023) and to study the time course of motion integration for complex signals.

## Synthesis

Reviewing Editor: David Schoppik, New York University Decisions are customarily a result of the Reviewing Editor and the peer reviewers coming together and discussing their recommendations until a consensus is reached. When revisions are invited, a fact-based synthesis statement explaining their decision and outlining what is needed to prepare a revision will be listed below. The following reviewer(s) agreed to reveal their identity: NONE. Note: If this manuscript was transferred from JNeurosci and a decision was made to accept the manuscript without peer review, a brief statement to this effect will instead be what is listed below.

The revised manuscript addresses all of the Reviewers' concerns and is stronger for the effort.
